# Heterostuctures of 4-(chloromethyl)phenyltrichlorosilane and 5,10,15,20-tetra(4-pyridyl)-21*H*,23*H*-porphine prepared on Si(111) using particle lithography: Nanoscale characterization of the main steps of nanopatterning

**DOI:** 10.3762/bjnano.9.112

**Published:** 2018-04-17

**Authors:** Phillip C Chambers, Jayne C Garno

**Affiliations:** 1 Department of Chemistry, Louisiana State University, 232 Choppin Hall, Baton Rouge, LA 70803, USA

**Keywords:** atomic force microscopy (AFM), nanostructures, particle lithography, porphyrin, self-assembly

## Abstract

Nanostructures of 4-(chloromethyl)phenyltrichlorosilane (CMPS) were used as a foundation to attach and grow heterostructures of porphyrins and organosilanes. A protocol was developed with particle lithography using steps of immersion in organosilane solutions to selectively passivate the surface of Si(111) with octadecyltrichlorosilane (OTS). A methyl-terminated matrix was chosen to direct the growth of CMPS nanostructures to fill the uncovered sites of Si(111) to enable spatial confinement of the surface reaction. Silica spheres with a diameter of 500 nm were used as a surface mask to prepare nanoscopic holes within the OTS matrix film. Next, the samples were immersed in solutions of CMPS dissolved in toluene or bicyclohexane. Nanostructures of CMPS formed within the nanoholes, to furnish spatially selective sites for binding porphyrins. The samples were then characterized with AFM to evaluate the height and morphology of the CMPS nanostructures that had formed within the nanoholes of OTS. The samples were then refluxed in a porphyrin solution for selective binding to produce heterostructures. The attachment of porphyrins was evidenced by increases in the height and width of the CMPS nanopatterns. The measurements of size indicate that multiple layers of porphyrins were added. Through each step of the surface reaction the surrounding matrix of OTS showed minimal areas of nonspecific adsorption. The AFM studies provide insight into the mechanism of the self-polymerization of CMPS as a platform for constructing porphyrin heterostructures.

## Introduction

The properties of porphyrins change inherently as a result of differences in macromolecular substituents, surface bonding mechanisms, surface orientation and coordinated metals [1]. The mechanisms by which porphyrins self-assemble on surfaces is complicated and is an area of active investigation [2–6]. The dynamics and advantages of supramolecular compounds of porphyrins within devices and in fabricated materials are relevant for molecular studies [7–8]. Properties of supramolecular films with porphyrins can be investigated with approaches such as non-linear optics [9], catalysis [10] and electronic measurements [11–13].

Investigations of porphyrins at interfaces have focused on elucidation of magnetic, photonic and electronic properties as well as the manner in which the molecules assemble on a surface. The adsorption of free-base tetraphenylporphyrin on Cu(111) was studied with scanning tunneling microscopy (STM) to evaluate the surface conformation and molecular geometry [14]. Individual molecules of nonplanar freebase and copper-metallated tetraphenyl porphyrins adsorbed on Cu(111) were investigated using frequency modulated noncontact AFM to resolve subtle differences in structure and conformation [15]. The submolecular structure of cobalt and copper phthalocyanines on gold substrates were resolved with STM by Lu et al. [16]. The differences in central metals were resolved for a mixed sample. The molecular orientation and molecular switching properties of a triple-decker sandwich complex of phthalocyanine compounds prepared on graphite was studied using STM by Lei et al. [17].

A method of photocatalytic lithography was reported for making porphyrin surface structures that were applied for preparing protein arrays [18–19]. The assembly of porphyrins at interfaces has been studied using layer-by-layer assembly that incorporates organosilane or organothiol monolayers to functionalize a surface to form multilayer films [2–3]. Dip-pen nanolithography was applied to pattern porphyrazines onto a polycrystalline gold surface to align horizontally or vertically with a surface orientation defined by the substituents [20]. The self-assembly of manganese *meso*-tetra(4-pyridyl)porphyrin on Cu(111) was studied using low temperature scanning tunneling microscopy (STM) and atomic force microscopy (AFM) to resolve molecular structures by Chen et al. [21]. A functionalized phthalocyaninato-polysiloxane was studied with STM on surfaces of highly oriented pyrolytic graphite (HOPG) by Samori et al. [22]. Photoelectronic devices of porphyrin polymers containing oligothienyl bridges were prepared as microscopic junction chips and as layered diodes by Shimadzu et al. [23]. Multiporphyrin assemblies have been proposed for molecular photonic devices due to the versatile physical properties [24].

Particle lithography is a patterning method that uses a surface mask of colloidal spheres to direct the deposition of molecules or other nanomaterials on surfaces. Particle lithography provides a way to produce millions of nanostructures with reproducible shapes, sizes and arrangements with organic thin films [25–26]. Particle lithography is also commonly referred to as nanosphere lithography (NSL) [27] and has been used to generate patterns of organic polymers [25,28–31], nanoparticles [32–35] and inorganic materials [36].

Experimental parameters such as the environmental conditions and solvent choice affect the density of organosilane thin films [37–38]. A model was proposed for the self-assembly of CMPS nanostructures formed within areas of nanoholes which subsequently grew to form multiple layers of CMPS through self-polymerization [37,39]. In a recent report, we have shown that changes in the parameters of temperature and solvent affect the growth of CMPS nanostructures prepared within a matrix film of organosilanes prepared with particle lithography [40].

In this investigation, the assembly and mode of growth for attaching 5,10,15,20-tetra(4-pyridyl)-21*H*,23*H*-porphine (H_2_TPyP) was studied as a model for binding porphyrins to 4-(chloromethyl)phenyltrichlorosilane (CMPS) nanostructures within a matrix film of octadecyltrichlorosilane (OTS). Multilayer structures of CMPS provide sites with benzyl halide for linking porphyrins to the surface at both the top as well as at the sides of nanopatterns. Particle lithography with successive steps of immersion reactions were used to prepare reactive surface sites to generate multicomponent nanostructures of porphyrins and organosilanes. With ex situ steps of particle lithography, the successive addition of molecules through chemical reactions in solution can be evaluated by measuring changes in the heights and morphology of nanostructures. Using high-resolution atomic force microscopy (AFM), surface changes can be subsequently characterized ex situ after each key step of the fabrication process.

## Results and Discussion

**Figure 1:**
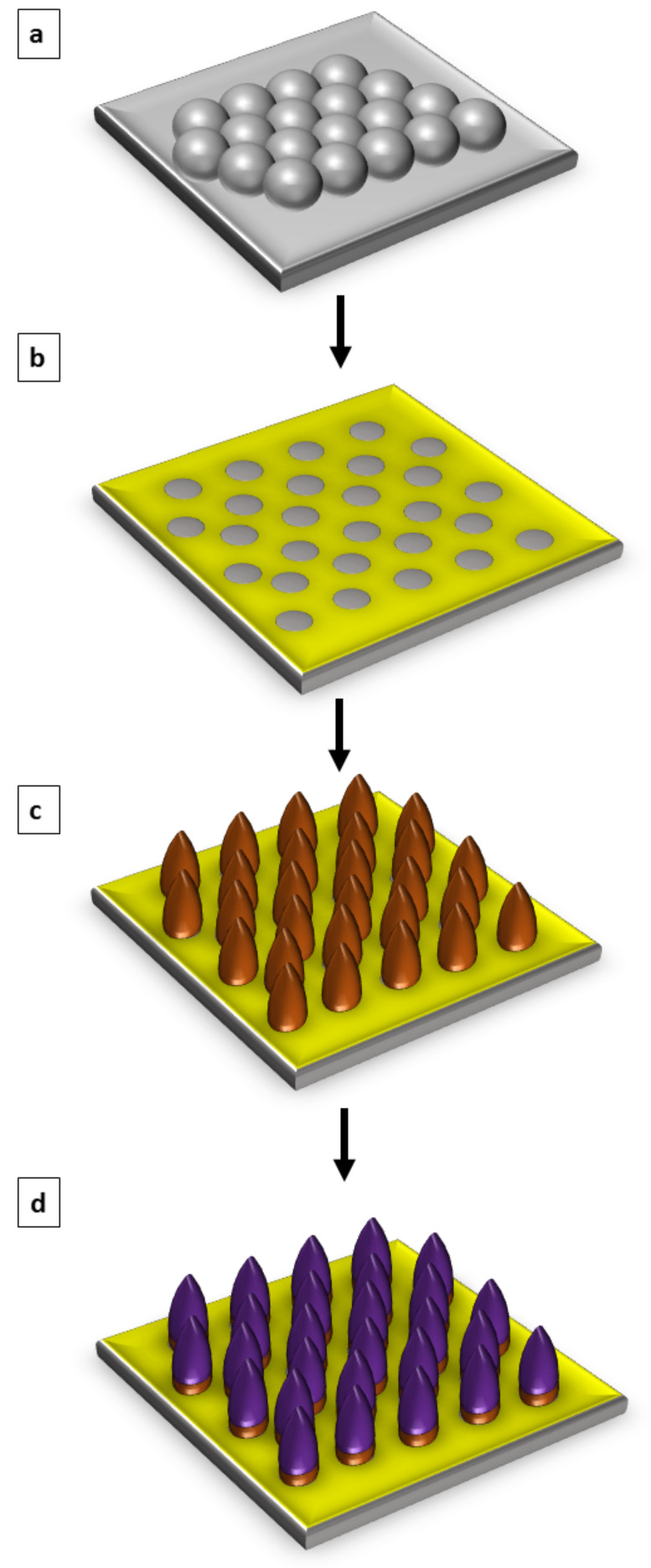
Steps for preparing nanopatterned CMPS-porphyrin heterostructures. (a) Monodisperse silica spheres were deposited on Si(111) to form a surface mask for particle lithography. (b) After immersion in OTS solution, the microspheres were rinsed away to reveal nanoholes of OTS. (c) With a second immersion reaction, nanodots of CMPS were produced. (d) Reaction with porphyrin produced taller heterostructures with spatial selectivity for the sites of CMPS nanodots.

An overview of the main steps for preparing nanostructures of H_2_TPyP within nanoholes of OTS is presented in Figure 1. The growth of nanopatterns and subsequent changes in surface morphology were characterized after each key step of sample preparation. A surface platform of nanoholes was generated in the first step by depositing silica spheres on a silicon substrate (Figure 1a). The masked surface was then immersed in a solution of OTS to form a methyl-terminated matrix film in between the silica spheres of the surface mask. The spheres were then removed with a washing step to produce a hexagonal pattern of nanoholes within the OTS film (Figure 1b), conforming to the arrangement of the surface mask. Samples with nanoholes within OTS were then placed in a solution of CMPS and either toluene or bicyclohexyl (BCH) for a selected amount of time to generate nanodots of CMPS (Figure 1c). Nanodots of CMPS formed selectively within the confined sites of nanoholes. The samples containing the CMPS nanodots were then refluxed in a solution of H_2_TPyP in ethanol and chloroform for 48 h to attach porphyrins (Figure 1d). Atomic force microscopy was used to characterize the resulting nanostructures after each step of the fabrication procedure. The attachment of the H_2_TPyP was confirmed by measuring changes in the width and height of nanostructures.

### Surface platform of nanoholes within a thin film of OTS on Si(111)

**Figure 2:**
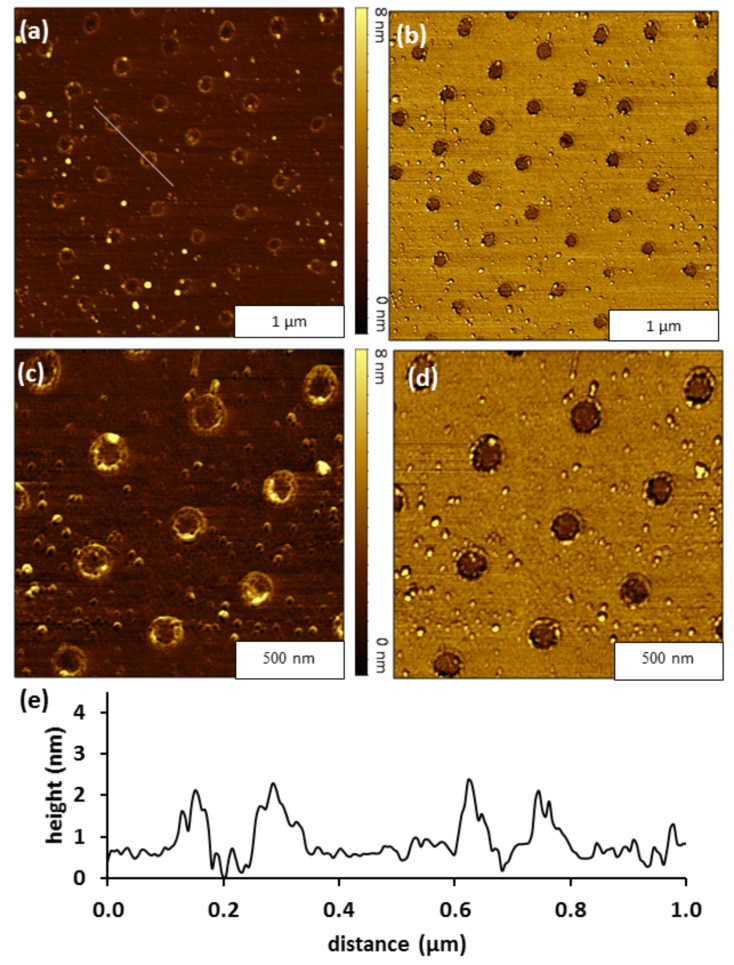
Nanoholes within a thin film of OTS. (a) Topography frame, 3 × 3 µm^2^; and simultaneously acquired (b) phase image. (c) Zoom-in topograph of nanoholes and corresponding (d) phase image. (e) Cursor profile for the line in (a).

Particle lithography with an immersion step was used to prepare nanoholes within a film of OTS. A topographic view of the nanoholes is shown in Figure 2a, with the simultaneously acquired phase image (Figure 2b).The ex situ images were acquired with tapping-mode AFM in air. The topograph reveals dark nanoholes within a surrounding OTS matrix (bright areas). The nanoholes formed a periodic arrangement throughout broad areas of microns. The distance between each nanohole corresponds to the 500 nm diameter of the Si spheres used to form a surface mask. The sites of nanoholes indicate the points of contact between the surface and the base of the Si spheres of the surface mask. The spheres protect small local areas from assembly of OTS. There are ≈40 nanoholes in Figure 2a, which scales to a surface density of 10^8^ nanoholes per cm^2^. Differences in tip–sample interactions are observed between the darker exposed nanoholes of Si(111) and the surrounding areas of the OTS matrix which are brighter, as revealed in the phase image presented in Figure 2b. The surface map of phase changes indicate the changes in the viscoelastic response that occurs between the tip and sample showing distinct differences in the interfacial chemistry of the uncovered silicon surface within the nanoholes versus the surrounding OTS matrix.

A closer look at the hexagonal arrangement of nanoholes is presented in Figure 2c and 2d. A few bright spots on the areas of OTS reveal trace contaminants that were not rinsed from the sample. The uniform color contrast observed in the phase image (Figure 2d) indicates that the nanoholes do not contain OTS. The approximate surface coverage of the OTS film measured 97%. The average thickness of the OTS monolayer was measured to be 0.7 nm. The measurements indicate submonolayer surface coverage relative to the ideal height (2.6 nm) of a densely packed OTS monolayer [25,41].

The nanoholes within OTS that were generated with particle lithography will serve as sites for further reactions with CMPS and H_2_TPyP to produce multicomponent nanostructures. Methyl-terminated OTS was chosen to passivate the silicon surface and to serve as a resist layer to accomplish spatial selectivity for surface reactions. The uncovered sites of Si(111) within the nanoholes expose hydroxyl groups for binding organosilanes such as CMPS.

### Preparation of CMPS nanodots

**Figure 3:**
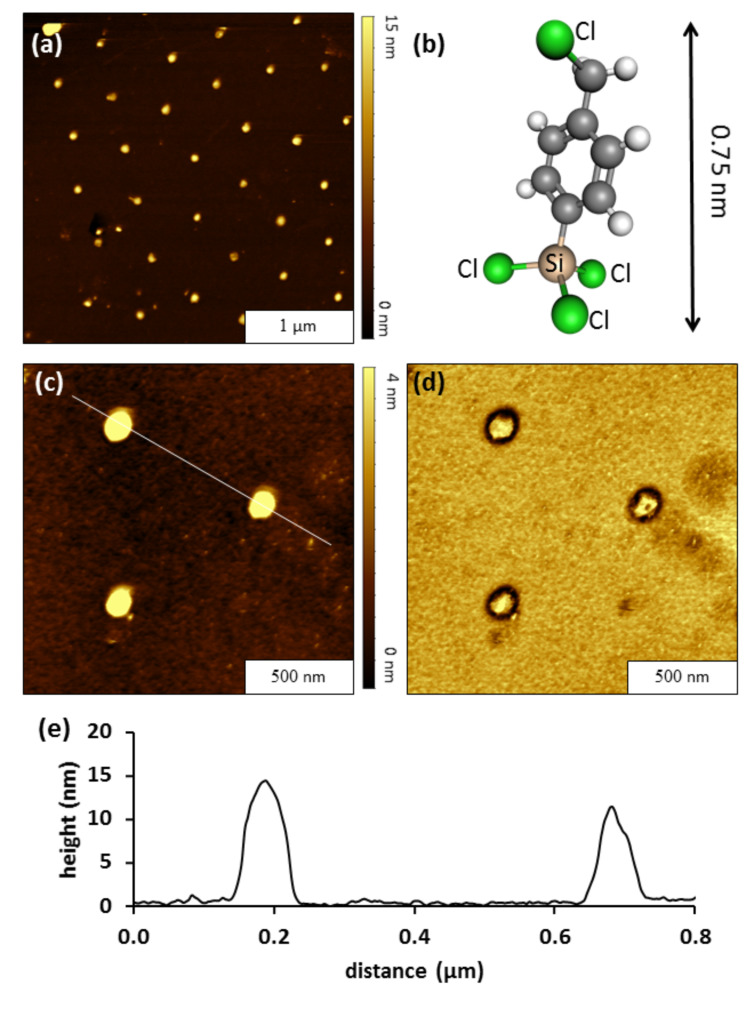
Nanodots of CMPS grown in a solution of BCH. (a) Topography image, 3 × 3 μm^2^; (b) structural model of CMPS. (c) High resolution (1.5 × 1.5 μm^2^) topography view of CMPS nanodots; (d) corresponding phase image. (e) Cursor profile for the line in (c).

The samples with nanoholes of Si(111) within an OTS resist were immersed in a solution of CMPS a to generate nanodots as reactive sites for further reaction steps with porphyrins. An example of the results for preparing nanodots of CMPS is shown in Figure 3. Nanodots grown in a solution of CMPS in BCH at 20 °C are shown in Figure 3a. The bright spots in Figure 3a are taller than the surrounding OTS matrix. There are about 35 CMPS nanodots visible in the 3 × 3 µm^2^ topography image in Figure 3a, which matches the surface density of OTS nanoholes. A ball-and-stick model of a CMPS molecule indicates a length of 0.75 nm in Figure 3b [38]. A close-up view of three nanodots are shown in zoom-in topography and phase images in Figures 3c and 3d. The heights and sizes of the nanodots are quite similar, without nonspecific attachment of contaminants in surrounding areas of the OTS resist film. There is a dark outline surrounding the nanodots that is apparent in the phase image (Figure 3d) which is attributable to differences in tip–surface response at the edges of the features versus the center areas of the nanostructures. The cursor profile in Figure 3e profiles the topography of two individual CMPS nanostructures that are shown in Figure 3c. The heights of the individual CMPS nanostructures traced in Figure 3e closely correspond to the overall average height measured under these reaction conditions. The height of the nanostructures measured 16 ± 3 nm (*n =* 35) above the OTS matrix, not including the depth of the nanoholes. The center-to-center spacing of each nanostructure measures 500 nm which matches to the diameter of the original surface mask of Si spheres. The areas with CMPS have self-polymerized to form multilayer nanostructures. The OTS resist confines the multilayer polymerization of CMPS to form within the exposed nanoholes of Si(111). Spatial confinement facilitated the growth of CMPS layers in the vertical direction, which was produced by cross-linking to form intramolecular siloxane bonds.

### Spatial selectivity for the preparation of heterostructures of CMPS and H_2_TPyP

**Figure 4:**
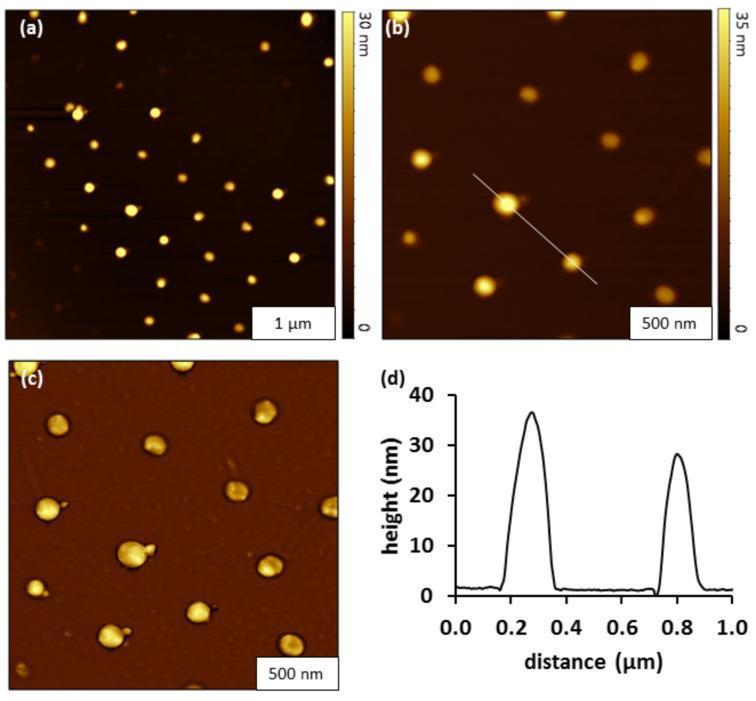
Heterostructures comprised of CMPS and H_2_TPyP porphyrin grown on Si(111) in BCH. (a) Topography image (4 × 4 μm^2^) of porphyrin nanostructures grown on CMPS nanodots. (b) Zoom-in topography view of porphyrin heterostructures; (c) simultaneously acquired phase image; (d) cursor profile for the line in (b).

Heterostructures of CMPS-porphyrin were generated by reacting nanopatterned substrates with CMPS nanodots in a solution of H_2_TPyP for 48 h at 100 °C (Figure 4). Characterizations with AFM were used to evaluate if H_2_TPyP bound selectively to the top of the patterns in a vertical growth process, or if the structures also became wider due to horizontal growth through binding at the sides of the nanodots. The surface placement of 45 CMPS-porphyrin nanostructures are shown within the 4 × 4 μm^2^ area of the topography image of Figure 4a. The hexagonal arrangement of nanopatterns is maintained with center-to-center spacing between nanostructures measuring 500 nm, as revealed in the close-up topography and phase views (Figure 4b and 4c). There is little nonspecific binding of adsorbates on the OTS matrix areas between the nanostructures, as shown in the phase map of Figure 4c. An example cursor profile that was traced across two of the taller heterostructures indicates that the heights range from 30 to 40 nm (Figure 4d). The average height of the CMPS-porphyrin heterostructures above the OTS matrix measured 24 ± 6 nm (*n =* 35), this is an increase of ≈8 nm from the size of the CMPS nanodots. (Detailed size analysis is provided in Supporting Information File 1, Figure S1)

The nanostructures of CMPS also showed growth in lateral dimensions after the addition of porphyrin. A comparison of the nanostructure surface coverage was conducted to evaluate lateral growth of the nanostructures before and after porphyrin addition. The percentage surface coverage of the nanostructures was measured from multiple sites on each of the surfaces (before and after porphyrin addition). The average surface coverage of the CMPS nanostructures was 2.3% of the surface evaluated for wider frames spanning 6 × 6 µm^2^. After porphyrin addition, the surface coverage increased to 4.6%. The difference in surface coverage was determined to be statistically significant at a 98% confidence level. The change in surface coverage is evidence of growth in the lateral dimensions of the CMPS-porphyrin nanostructures and confirms that porphyrin has attached to the CMPS nanodots.

### Proposed model for constructing heterostructures of CMPS and H_2_TPyP

**Figure 5:**
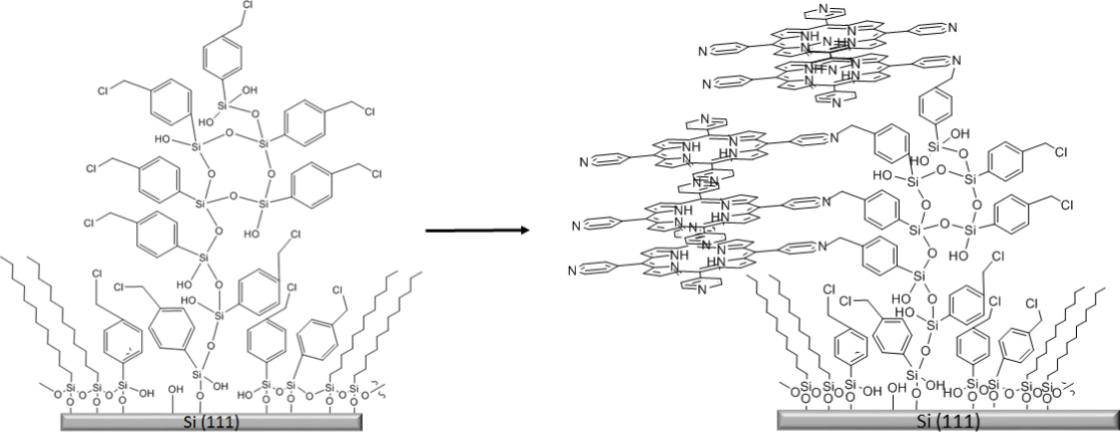
Model for the self-assembly of CMPS-porphyrin heterostructures within an OTS matrix layer.

The reaction for producing CMPS nanodots is driven by hydrolysis and condensation reactions that promote the vertical growth of CMPS through crosslinking siloxane bonds [40]. We did not observe evidence of branching or growth in lateral dimensions for CMPS nanodots, the growth was directed in the vertical direction to create taller nanostructures. However, the nanodot structures became taller and wider after reaction with H_2_TPyP to form heterostructures. This indicates that 3D growth takes place in the nanostructure assembly through the addition of H_2_TPyP. The increases in the height of nanostructures are attributable in part to coplanar interactions between porphyrin macrocycles leading to π–π stacking, as well as by a vertical orientation of the molecules attached to the topmost areas of CMPS nanodots. A possible model of how growth of H_2_TPyP occurs in both vertical and horizontal directions is presented in Figure 5. Nitrogen containing pyridyl groups that are substituents of the porphyrin participate in the replacement of the benzyl halide that is exposed at the outer regions of the CMPS nanostructure. Previous studies without nanopatterning steps investigated the application of CMPS as a coupling layer for the addition of H_2_TPyP to produce a porphyrin thin film [2–3]. Multilayer films are stabilized by siloxane bonds that form the backbone of the CMPS linker, as well as by weaker π–π interactions between the benzene rings of CMPS and the porphyrin macrocycles.

## Conclusion

Particle lithography was successfully applied to generate nanopatterns to determine the surface placement of porphyrin-CMPS heterostructures. Nanoholes were used to spatially direct the fabrication of complex nanostructures on Si(111) using multiple steps of immersion reactions combined with particle lithography. Periodic arrangements of heterostructures of CMPS-H_2_TPyP heterostructures were generated through a multistep layer-by-layer assembly process. A film of methyl-terminated OTS provided an effective resist for preventing nonspecific adsorption or reactions on areas between nanopatterns during successive chemical steps. Nanodots of CMPS were used as a linker for binding porphyrins to the surface. The changes in surface morphology were examined after each step using ex situ AFM studies. A model was proposed for attachment of H_2_TPyP to CMPS nanodots with growth observed in both the vertical and lateral directions. Particle lithography provides a practical tool for evaluating surface growth and changes for multistep chemical reactions on surfaces.

## Experimental

### Materials and reagents

The porphyrin selected, 5,10,15,20-tetra(4-pyridyl)-21*H*,23*H*-porphine (H_2_TPyP) (97%) was obtained from Sigma-Aldrich (St. Louis, MO). Anhydrous ethanol (200 proof) was purchased from Pharmco-Aaper (Shelbyville, KY). Chloroform, (HPLC grade) was obtained from Avantor Performance Materials (Center Valley, PA). Octadecyltrichlorosilane (97%) and (*p*-chloromethyl)phenyltrichlorosilane (95%) were purchased from Gelest (Morrisville, PA).

### Preparation of OTS nanoholes within an OTS matrix film

Particle lithography was used to prepare nanoholes within a thin film of octadecyltrichlorosilane (OTS) on Si(111). Silicon wafers (Ted Pella Inc. Redding, California) were rinsed with water and cleaned in piranha solution (3:1 sulfuric acid to hydrogen peroxide) for 1.5 h to remove surface contaminants. Caution: this solution is highly corrosive and should be handled carefully. The substrates were then rinsed with ultrapure water and dried under nitrogen. After drying, 10 µL of monodisperse silica microspheres in water was deposited on the clean silicon substrates and dried in air to produce a surface film of Si spheres. The substrate and dried microspheres were placed in an oven at 150 °C for 20 h. The annealing heating step was used to temporarily solder the silica microspheres to the silicon surface so that the beads would not be displaced in solution. The substrates containing the silica microspheres masks were then removed from the oven and placed in a 0.1% (v/v) solution of OTS in toluene for 5 h. The samples were then rinsed with ethanol and water with successive sonication in ethanol, ultrapure water, and chloroform. A rinsing and sonication step was used to fully remove the spheres from the surface. The samples were then dried under argon and characterized with AFM.

### Preparation of CMPS nanostructures

The samples with nanoholes within OTS were immersed in a 0.6% solution of CMPS in bicyclohexane (Sigma-Aldrich, St. Louis, MO) for 30 min. After the immersion step, samples were rinsed and sonicated in ethanol and chloroform. The samples were then dried under nitrogen and subsequently characterized with AFM.

### Preparation of heterostructures of CMPS and porphyrins

The samples with CMPS nanodots within an OTS matrix were immersed in a solution of H_2_TPyP in ethanol (1.8 mM) and chloroform (ratio of 1:9 respectively) and refluxed at 90 °C for 48 h. The samples were removed and rinsed with ethanol, then sonicated in chloroform and ethanol for 5 min. The sonication step was repeated 4 times and then the samples were dried under nitrogen.

### Atomic force microscopy

Samples were characterized using a model 5500 atomic force microscope (Keysight Technologies, Santa Rosa, CA). Images of samples were acquired using tapping-mode in ambient air. Silicon nitride tips that have force constants ranging from 10 to 30 N/m, and resonance frequencies ranging from 265 to 280 kHz (Nanosensors, Neuchatel, Switzerland) were used for AFM studies. Digital images were processed using the open source software Gwyddion, which is supported by the Czech Metrology Institute [42].

## Supporting Information

A histogram of the height measurements for the nanostructures for Figure 4 is provided in the Supporting Information.

File 1 Size distribution for the heights measured for heterostructures of CMPS and H_2_TPyP.
