# Supplementary material for: Heterostuctures of 4-(chloromethyl)phenyltrichlorosilane and 5,10,15,20-tetra(4-pyridyl)-21H,23H-porphine prepared on Si(111) using particle lithography: Nanoscale characterization of the main steps of nanopatterning
Source: Beilstein J Nanotechnol. 2018 Apr 17;9:1211–9. doi: 10.3762/bjnano.9.112 (PMC5942378; doi:10.3762/bjnano.9.112)
Supplement: File 1 — Size distribution for the heights measured for heterostructures of CMPS and H2TPyP. [file Beilstein_J_Nanotechnol-09-1211-s001.pdf]

Supporting Information

for

Heterostuctures of 4-(chloromethyl)phenyltrichlorosilane and 5,10,15,20-tetra(4-pyridyl)-21*H*,23*H*-porphine prepared on Si(111) using particle lithography: Nanoscale characterization of the main steps of nanopatterning

Phillip C. Chambers and Jayne C. Garno[*,§]

Address: Department of Chemistry, Louisiana State University, 232 Choppin Hall, Baton Rouge, LA 70803, USA

Email: Jayne C. Garno - jgarno@lsu.edu

*Corresponding author

[§]Phone: +1-225-578-8942, Fax: +1-225-5783458

Size distribution of the heights measured for heterostructures of CMPS and H_2TPyP.

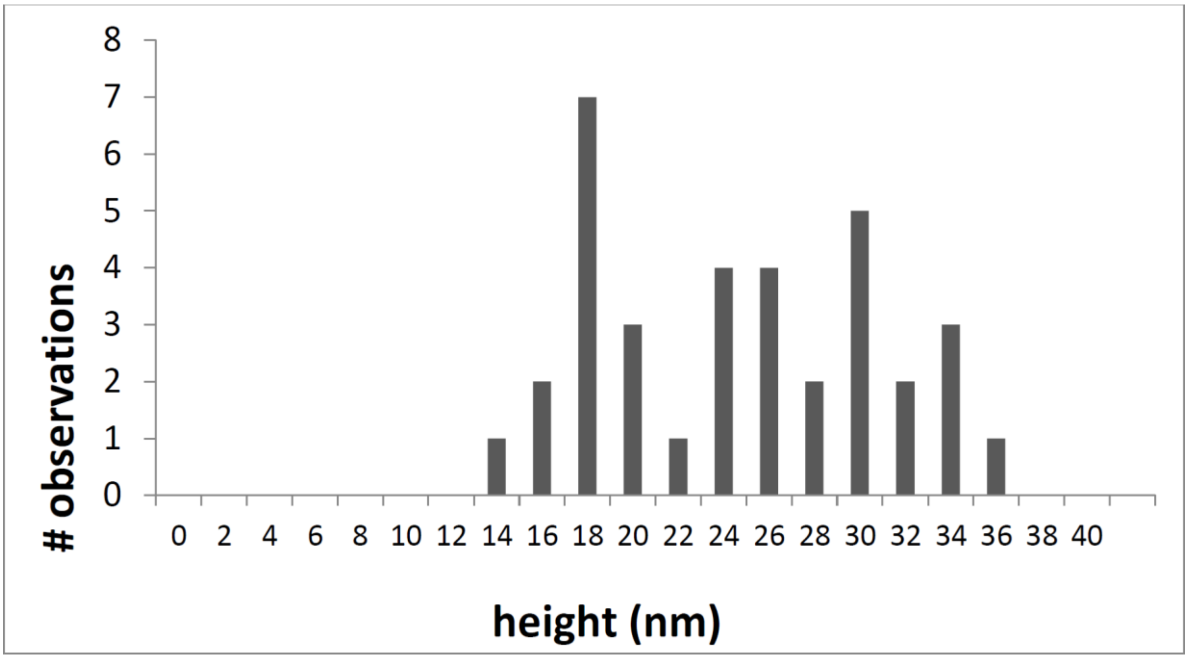

Figure S1: Range of heights for nanostructures of H_2TPyP formed on nanodots of CMPS within a matrix film of OTS formed in BCH. The measurements indicate the height above the surrounding OTS matrix.
